# Probing the Interactions of 31 Mycotoxins with Xanthine Oxidase: Alternariol, Alternariol-3-Sulfate, and α-Zearalenol Are Allosteric Inhibitors of the Enzyme

**DOI:** 10.3390/toxins15040250

**Published:** 2023-03-29

**Authors:** Orsolya Balázs, Ágnes Dombi, Balázs Zoltán Zsidó, Csaba Hetényi, Róbert György Vida, Miklós Poór

**Affiliations:** 1 Department of Pharmacology, Faculty of Pharmacy, University of Pécs, Rókus u. 2, H-7624 Pécs, Hungary; 2 Department of Pharmaceutics and Central Clinical Pharmacy, Faculty of Pharmacy, University of Pécs, H-7624 Pécs, Hungary; 3 Unit of Pharmacoinformatics, Department of Pharmacology and Pharmacotherapy, Medical School, University of Pécs, Szigeti út 12, H-7624 Pécs, Hungary; 4 Food Biotechnology Research Group, János Szentágothai Research Centre, University of Pécs, Ifjúság útja 20, H-7624 Pécs, Hungary

**Keywords:** mycotoxins, xanthine oxidase, alternariol, alternariol-3-sulfate, α-zearalenol, enzyme inhibition

## Abstract

Mycotoxins are frequent toxic contaminants in foods and beverages, causing a significant health threat. Interactions of mycotoxins with biotransformation enzymes (e.g., cytochrome P450 enzymes, sulfotransferases, and uridine 5′-diphospho-glucuronosyltransferases) may be important due to their possible detoxification or toxic activation during enzymatic processes. Furthermore, mycotoxin-induced enzyme inhibition may affect the biotransformation of other molecules. A recent study described the strong inhibitory effects of alternariol and alternariol-9-methylether on the xanthine oxidase (XO) enzyme. Therefore, we aimed to test the impacts of 31 mycotoxins (including the masked/modified derivatives of alternariol and alternariol-9-methylether) on XO-catalyzed uric acid formation. Besides the in vitro enzyme incubation assays, mycotoxin depletion experiments and modeling studies were performed. Among the mycotoxins tested, alternariol, alternariol-3-sulfate, and α-zearalenol showed moderate inhibitory actions on the enzyme, representing more than tenfold weaker impacts compared with the positive control inhibitor allopurinol. In mycotoxin depletion assays, XO did not affect the concentrations of alternariol, alternariol-3-sulfate, and α-zearalenol in the incubates; thus, these compounds are inhibitors but not substrates of the enzyme. Experimental data and modeling studies suggest the reversible, allosteric inhibition of XO by these three mycotoxins. Our results help the better understanding of the toxicokinetic interactions of mycotoxins.

## 1. Introduction

Mycotoxins are secondary metabolites of molds. Mycotoxin contamination in foods and beverages causes significant health threats worldwide [1]. Their typically high thermal stability and frequent occurrence in the food chain make the removal of mycotoxins very challenging [2,3]. Biotransformation enzymes (such as cytochrome P450 enzymes, sulfotransferases, and uridine 5′-diphospho-glucuronosyltransferases) play important roles in the toxic activation or detoxification of mycotoxins; in addition, the mycotoxin-induced inhibition of these enzymes may affect the biotransformation of other molecules [4].

The *Alternaria* mycotoxins alternariol (AOH) and alternariol-9-methylether (AME), as well as their masked (glucoside) and modified (sulfate) derivatives, typically contaminate tomato products (Table 1) [5,6,7]. The sulfate metabolites, such as alternariol sulfate (AS) and alternariol monomethyl ether sulfate (AMS), are also produced by sulfotransferases in mammals [8,9].

Aflatoxins, sterigmatocystin (STC), and cyclopiazonic acid (CPA) are usually produced by *Aspergillus* molds (Table 1) [10,11,12,13]. Aflatoxin B1 (AFB1) is the most common and most toxic aflatoxin derivative, whereas aflatoxin M1 (AFM1) frequently contaminates milk [11]. STC can also be considered an intermediate product in aflatoxin biosynthesis [12]. Citrinin (CIT), ochratoxins, and patulin (PAT) are typical products of *Aspergillus* and *Penicillium* fungi (Table 1) [1,14,15]. Furthermore, dihydrocitrinone (DHC) is the main metabolite of CIT in the urine of mammals [16]. Among ochratoxins, ochratoxin A (OTA) is the most common contaminant; however, ochratoxin B (OTB) and ochratoxin C (OTC) also appear in the food chain (e.g., in some wines) [17,18,19].

*Fusarium* strains are responsible for the production of several mycotoxins, including beauvericin (BEA), deoxynivalenol (DON or vomitoxin), fumonisin B1 (FB1), T-2 toxin (T2), and zearalenone (ZEN) (Table 1) [20]. During the biotransformation of ZEN in mammals, reduced and conjugated metabolites are formed. Reduced derivatives are zearalenols (ZEL), zearalanone (ZAN), and zearalanols (ZAL); some of these metabolites (e.g., α-ZEL and α-ZAL) exert much stronger xenoestrogenic effects than the parent mycotoxin [21,22]. In mammals, uridine 5′-diphospho-glucuronosyltransferase and sulfotransferase enzymes can produce glucuronide (e.g., zearalenone-14-glucuronide) and sulfate (e.g., zearalenone-14-sulfate (Z14S)) conjugates, respectively [21]. In addition, masked/modified derivatives also appear in cereals (e.g., wheat, maize, and barley), such as Z14S or zearalenone-14-glucoside (Z14Glz) [21].

Xanthine oxidoreductase is a 300 kDa homodimer protein containing molybdenum cofactor, flavin adenine dinucleotide (FAD) site, and Fe_2_S_2_ sites [23]. The enzyme is important in purine catabolism because it catalyzes the transformation of hypoxanthine to xanthine and then xanthine to uric acid [24]. Xanthine oxidoreductase designation means two interconvertible forms of the same enzyme, including xanthine dehydrogenase and xanthine oxidase (XO). Xanthine dehydrogenase can be reversibly or irreversibly converted to XO [25]. Typically, xanthine dehydrogenase is intracellularly presented, while XO is dominant in the extracellular water space [23]. High uric acid levels cause hyperuricemia which may result in the development of gout, cardiovascular diseases, and metabolic syndrome [26]. In addition, XO generates superoxide anion radicals and hydrogen peroxide, which may also be involved in the unpleasant impacts of high uric acid formation [27]. Allopurinol, a potent inhibitor of XO, is commonly used to treat hyperuricemia and gout [28]. Furthermore, XO is also an important enzyme in the biotransformation of certain drugs, such as 6-mercaptopurine (used in the treatment of cancer and autoimmune diseases) [29].

Previous studies demonstrated that aflatoxins and ochratoxins could cause the development of gout in certain animals [30]. AFB1-contaminated diet (for 14 days and 21 days) increased XO activity and uric acid levels in the serum of fish [31]. In rats, chronic exposure to ZEN significantly elevated XO activity in the liver and kidneys [32]. The upregulation of xanthine oxidoreductase was observed in the liver and the jejunum of chickens after three weeks of DON-contaminated feeding [33]. In contrast, fumonisins (FB1 + FB2) alone and in combination with DON (15 days of exposure) decreased the expression of xanthine dehydrogenase in the jejunum of broiler chickens [34]. These results suggest that certain mycotoxins may be able to increase the expression and/or the activity of xanthine oxidoreductase.

Typically, less information is available regarding the inhibitory effects of mycotoxins on XO. In a recent study, Fan et al. described that AOH (IC_50_ = 0.2 μM) and AME (IC_50_ = 0.5 μM) are strong inhibitors of XO, where these mycotoxins showed approximately tenfold higher inhibitory potency compared with allopurinol [35]. Considering these results and the relatively low acute toxicity of AOH and AME, Fan et al. suggested that AOH may be a potential lead compound in the development of new potent XO inhibitors [35]. Urolithins (colon metabolites of ellagitannins) are considered barely toxic compounds [36]. They have very similar chemical structure to *Alternaria* mycotoxins. In the study of Fan et al. [35], urolithins examined were much weaker inhibitors of XO than AOH or AME. In another paper, considerably weaker inhibitory actions of AOH (IC_50_ = 15.5 μM) and AME (IC_50_ = 60.5 μM) were described regarding the XO enzyme [37]. In addition, the consumption of wheat grain contaminated with *Alternaria* spp. did not affect XO activity in broiler chickens [38].

Importantly, XO is a major component in bovine milk [39], which is commonly contaminated with certain mycotoxins (e.g., AFM1, OTA, ZEN, and α-ZEL) [40]. Thus, mycotoxin–XO interactions can also be important from this point of view.

**Figure 1:**
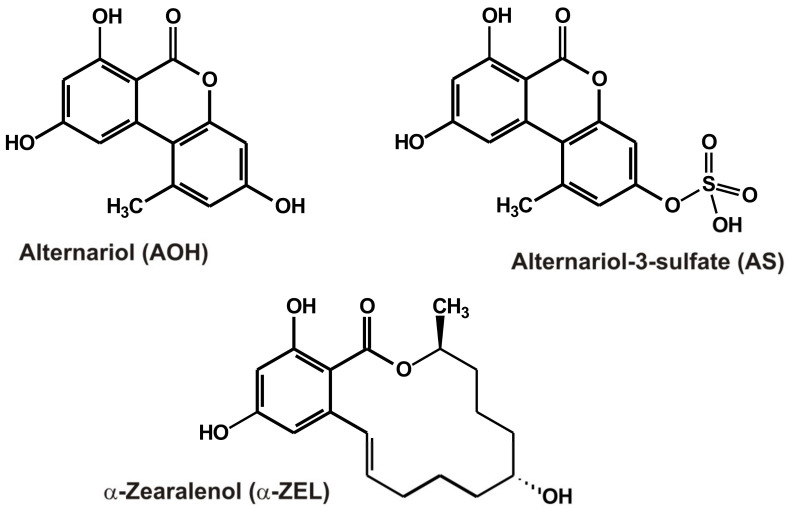
Chemical structures of mycotoxins alternariol, alternariol sulfate, and α-zearalenol.

In this work, the interactions of 31 mycotoxins with the XO enzyme were examined by applying in vitro enzyme assays and modeling studies. We planned to test the effects of AOH, AME, and their masked/modified derivatives (AS, AG, AMS, and AMG) on XO-catalyzed uric acid formation to confirm the previously reported data and to find potentially stronger inhibitors among AOH derivatives. In addition, we also investigated the impacts of 25 other mycotoxins, including AFB1, AFB2, AFG1, AFG2, AFM1, STC, CPA, CIT, DHC, OTA, OTB, OTC, PAT, BEA, DON, FB1, T2, ZEN, α-ZEL, β-ZEL, ZAN, α-ZAL, β-ZAL, Z14S, and Z14Glz (Table 1). The potential inhibitory actions of mycotoxins on XO, the reversibility of their inhibitory effects, and the XO-induced mycotoxin depletion were assessed. Experimental results and molecular modeling studies suggest the moderate, allosteric inhibition of XO by AOH, AS, and α-ZEL (Figure 1).

## 2. Results and Discussion

### 2.1. Inhibitory Effects of Mycotoxins on Xanthine Oxidase Enzyme

**Figure 2:**
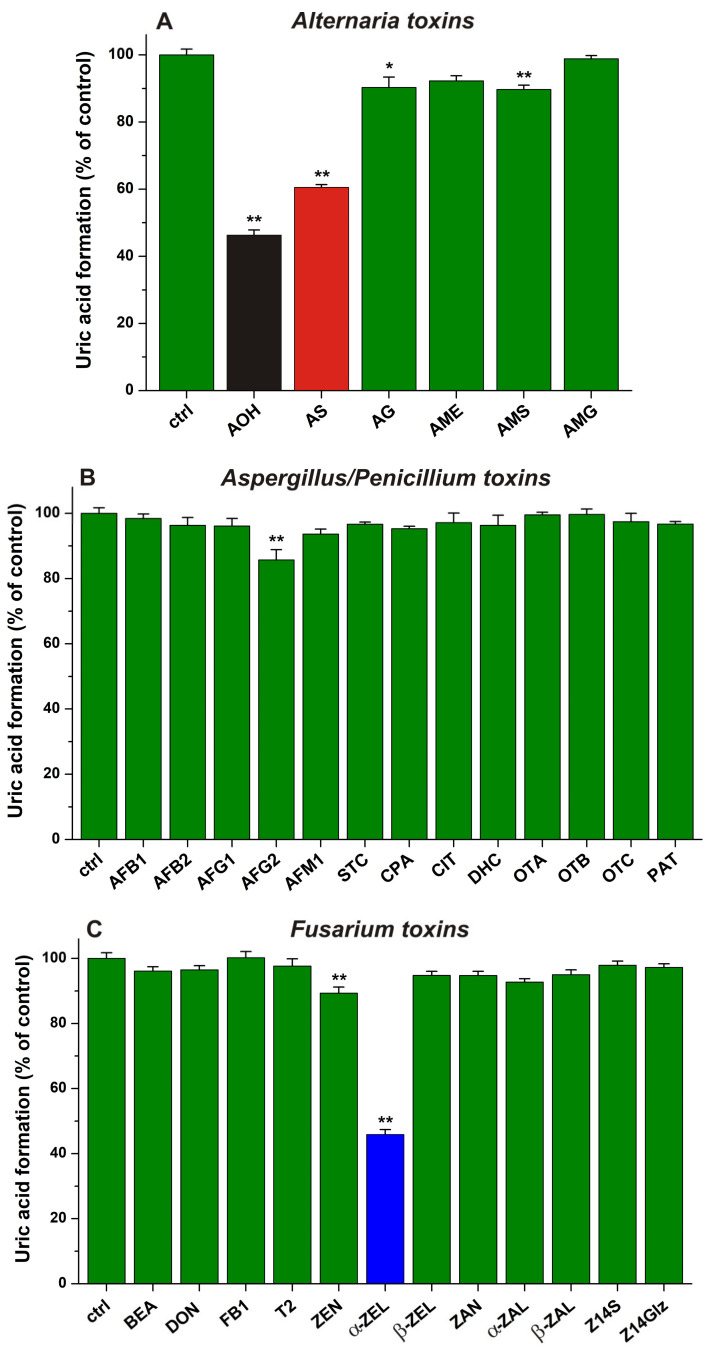
Effects of *Alternaria* (**A**), *Aspergillus*/*Penicillium* (**B**), and *Fusarium* (**C**) mycotoxins (each 20 μM) on XO-catalyzed uric acid formation (substrate concentration: 5 μM; incubation: 5 min; n = 3; * *p* < 0.05 and ** *p* < 0.01). We started the reaction with the addition of XO. The highest solvent concentrations applied (0.4% ethanol or 0.4% DMSO) did not affect the XO-catalyzed uric acid formation (101.1 ± 1.2% in the presence of 0.4% ethanol; 99.2 ± 1.9% in the presence of 0.4% DMSO).

The impacts of 20 μM mycotoxin concentrations were tested to evaluate the potential inhibitory effects of mycotoxins on XO-catalyzed xanthine oxidation. Results are demonstrated in Figure 2, where *Alternaria*, *Aspergillus*/*Penicillium*, and *Fusarium* toxins are presented in three separate panels.

AOH, AS, AG, and AMS induced a statistically significant decrease in metabolite formation, while AME and AMG caused only slight or no effects, respectively (Figure 2A). Among the *Alternaria* mycotoxins tested, AOH and AS showed the strongest inhibitory actions (resulting in a 54% and 40% decrease in uric acid formation, respectively). AG and AMS induced approximately 10% inhibition; therefore, these mycotoxins can be considered weak inhibitors of XO. Interestingly, our results suggest negligible inhibition of the enzyme by AME, while in a previous report, AME and AOH showed similarly strong inhibitory effects [35]. Furthermore, AS caused a slightly weaker impact vs. AOH (Figure 2A). This result is in accordance with our previous observation that quercetin-3′-sulfate was also a similarly strong inhibitor of XO than the parent flavonoid quercetin [41].

In Figure 2B, the impacts of *Aspergillus*/*Penicillium* mycotoxins are presented: AFB1, AFB2, AFG1, AFM1, STC, CPA, CIT, DHC, OTA, OTB, OTC, and PAT did not affect the XO-catalyzed metabolite formation. AFG2 showed a statistically significant but weak inhibitory action (14%) on XO (Figure 2B).

Among the *Fusarium* mycotoxins, BEA, DON, FB1, T2, β-ZEL, ZAN, α-ZAL, β-ZAL, Z14S, and Z14Glz did not influence the XO-catalyzed uric acid formation (Figure 2C). However, ZEN induced a small decrease (10%), while α-ZEL caused a marked (54%) decrease in metabolite production. To the best of our knowledge, the inhibitory actions of ZEN and α-ZEL on the XO enzyme have not been previously reported. Nevertheless, in a recent in vivo study, chronic exposure to ZEN significantly elevated XO activity in the liver and kidneys of rats [32]. These observations suggest that ZEN and/or its metabolites may affect the expression of XO, which mechanism may overwrite the direct inhibitory actions noticed for ZEN and α-ZEL in the current study.

**Figure 3:**
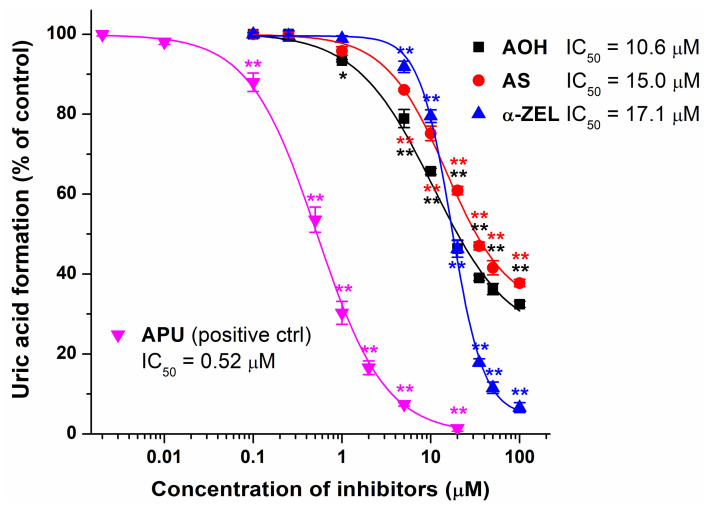
Concentration-dependent inhibitory actions of AOH, AS, α-ZEL, and allopurinol (APU, positive control) on XO-catalyzed xanthine oxidation (substrate concentration: 5 μM; incubation: 5 min; n = 3; * *p* < 0.05 and ** *p* < 0.01). We started the reaction with the addition of XO. The 50 μM and 100 μM AOH and AS samples contained 0.5% (uric acid formation = 99.0 ± 1.1%) and 1.0% (uric acid formation = 91.5 ± 2.1%) DMSO, respectively. The 50 μM and 100 μM α-ZEL samples contained 1.0% (uric acid formation = 96.8 ± 1.8%) and 2.0% (uric acid formation = 95.8 ± 1.3%) ethanol, respectively. Since the highest levels of ethanol (2.0%) and DMSO (1.0%) applied caused some inhibitory effects on the enzyme, the metabolite formation in the presence of 100 μM of AOH, AS, and α-ZEL was calculated compared to the corresponding solvent controls. The highest concentration APU sample (20 μM) tested contained 0.4% DMSO, which did not affect metabolite formation (99.2 ± 1.9%).

The concentration-dependent inhibitory effects of AOH, AS, and α-ZEL on XO were also evaluated compared to the positive control allopurinol. Even 1 μM of AOH induced statistically significant (*p* < 0.05) inhibition, while each compound caused a significant (*p* < 0.01) decrease in uric acid formation at 5 μM concentration (Figure 3). At their lower levels (1, 5, and 10 μM), both AOH and AS showed stronger inhibitory effects than α-ZEL; however, at 20 μM, AOH and α-ZEL produced similar impacts. In addition, at their highest concentrations tested (35, 50, and 100 μM), α-ZEL caused much stronger inhibition than AOH and AS (Figure 3). In the presence of 100 μM of mycotoxins, AOH, AS, and α-ZEL induced a 68%, 62%, and 93% decrease in metabolite formation, respectively.

Based on sigmoidal fitting, the IC_50_ values of AOH, AS, and α-ZEL were 10.6 μM, 15.0 μM, and 17.1 μM, respectively. Nevertheless, it is important to note that α-ZEL can produce close to complete inhibition at high concentrations; while for AOH and AS, we observed the lower plateau of the sigmoid curve around 35% metabolite formation. In the same assay, allopurinol showed much stronger inhibition on XO-catalyzed xanthine oxidation; its IC_50_ value was 0.5 μM. These data demonstrated that AOH, AS, and α-ZEL are approximately 20- to 35-fold weaker inhibitors of XO compared with the positive control allopurinol. Therefore, our results do not support the previous study of Fan et al. [35], which suggested that AOH and AME are highly potent inhibitors of XO. This is surprising because, in our earlier studies with flavonoids and XO [41,42], we determined similar IC_50_ values to other research groups. However, in regard to their inhibitory actions on XO enzyme, another study suggested higher IC_50_ values of AOH (IC_50_ = 15.5 μM) and AME (IC_50_ = 60.5 μM) [37]. These data are in accordance with our findings, showing a similar IC_50_ value of AOH determined in the current study and explaining why we did not notice relevant inhibitory effect of AME (20 μM). Nevertheless, the consumption of wheat grain contaminated with *Alternaria* spp. did not affect XO activity in broiler chickens [38], suggesting the minor in vivo relevance of the moderate inhibitory actions of *Alternaria* mycotoxins on XO.

**Figure 4:**
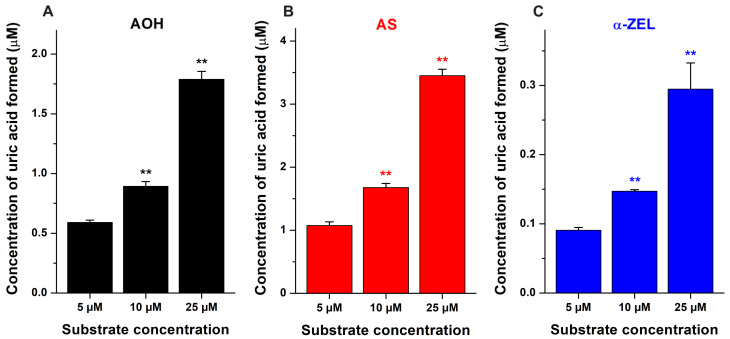
XO-catalyzed uric acid formation in the presence of 50 μM AOH (**A**), 50 μM AS (**B**) or 50 μM α-ZEL (**C**), with increasing concentrations of xanthine (5–25 μM; incubation: 5 min; n = 3). The enzyme was preincubated (10 min, 700 rpm, and 37 °C) with the mycotoxins, after which we started the reaction with the addition of the substrate (xanthine). Metabolite formation in the presence of 10 μM and 25 μM xanthine was compared to the product formation determined with 5 μM substrate concentration (** *p* < 0.01).

In the following experiment, we examined the reversibility of mycotoxin-induced XO inhibition. Therefore, the XO enzyme was preincubated with AOH, AS, or α-ZEL (each 50 μM) for 10 min, then we started the reaction with the addition of the substrate (final concentrations: 5, 10, or 25 μM). Similar to the previous experiments, the reaction was stopped after 5 min incubation. In a concentration-dependent fashion, the higher levels of the substrate significantly increased the XO-catalyzed uric acid formation (Figure 4). These results demonstrate that AOH, AS, and α-ZEL are reversible inhibitors of XO.

AOH, ZEN, and their derivatives appear in the circulation (and likely in tissues), typically at nanomolar concentrations [8,21]. However, the therapeutic plasma concentrations of allopurinol (competitive inhibitor and false substrate of XO) and its metabolite oxipurinol (pseudo-irreversible inhibitor of XO) are approximately 40 μM together [43]. In addition, allopurinol and oxipurinol are highly potent inhibitors of XO. Considering the significantly higher IC_50_ values of AOH, AS, and α-ZEL (IC_50_ = 10.6–17.1 μM) compared with allopurinol (IC_50_ = 0.5 μM) as well as the much lower concentrations of these mycotoxins in the body, it is very unlikely that AOH, AS, or α-ZEL can induce a clinically relevant decrease in uric acid levels. Based on these data, it is also reasonable to hypothesize that AOH, AS, and α-ZEL are not able to disrupt the XO-mediated biotransformation of 6-mercaptopurine or other drugs (e.g., azathioprine). Considering the previous observations in animal studies [30,31,32,33,34], some mycotoxins may be able to induce the increased expression of XO, which likely have more in vivo relevance than the inhibitory effects noticed in the current study.

### 2.2. Mycotoxin Depletion Assays

AOH, AS, and α-ZEL showed significant inhibitory effects on XO; therefore, we examined whether these mycotoxins are simply inhibitors or if they are also substrates of the enzyme. AOH, AS, and α-ZEL were incubated for 0 min, 30 min, and 60 min in the presence of the same amount of XO (0.0012 U/mL) used in the enzyme inhibition studies. Nevertheless, we did not see any changes in the concentrations of these mycotoxins (Table 2), suggesting that XO is not involved in the biotransformation of AOH, AS, and α-ZEL.

XO is a major constituent of bovine milk [39]. The simultaneous presence of XO and certain mycotoxins (e.g., AFM1, OTA, ZEN, and α-ZEL) [40] in milk also makes important mycotoxin–XO interactions, including the potential XO-mediated biotransformation. Our data suggest that XO is not involved in the metabolism of AOH, AS, and α-ZEL. Nevertheless, α-ZEL commonly contaminates bovine milk. Therefore, it is reasonable to hypothesize that α-ZEL appears in milk partly in XO-bound form, which may affect its release and absorption from the gastrointestinal tract.

### 2.3. Modeling Studies

**Figure 5:**
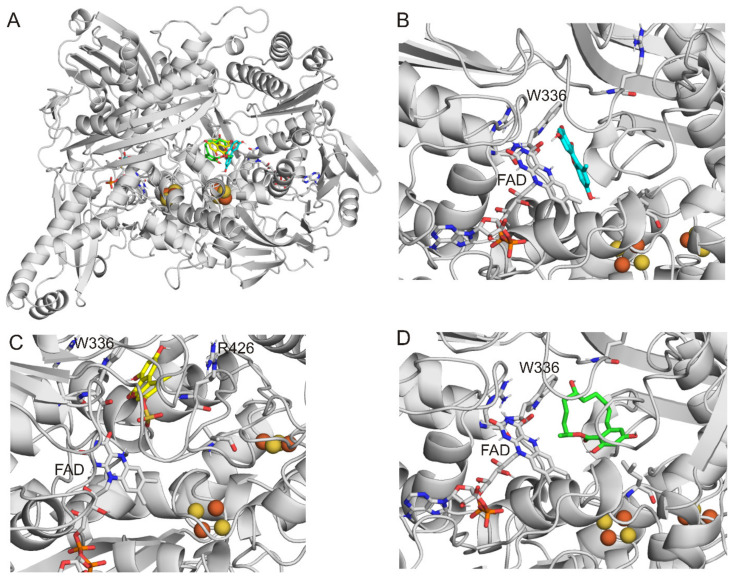
Binding modes of AOH (teal sticks), AS (yellow sticks), and α-ZEL (green sticks) in the alternative binding pocket of XO enzyme (**A**). The top 8th-ranked binding mode of AOH (**B**), the 1st-ranked binding mode of AS (**C**), and the 2nd-ranked binding mode of α-ZEL (**D**). XO is represented with a grey cartoon, FAD and molibdopteroate are demonstrated with grey sticks, and the Fe_2_S_2_ cluster is shown with spheres.

During the 300 blind docking runs in total, none of the ligands bound to the binding pocket of xanthine [44]. Nevertheless, in the best ranks for each ligand tested, another binding pocket was found (Figure 5A), which was originally described by Kuwabara et al. [45]. AOH (8th-rank binding mode; Figure 5B), AS (1st-rank binding mode; Figure 5C), and α-ZEL (2nd-rank binding mode; Figure 5D) interact with W336, which amino acid is located in an alternative binding pocket far from the xanthine binding site. Furthermore, modeling studies suggest the interaction of AOH and AS with K433. Among the three mycotoxins examined, AS has the most favorable ΔG_binding_ value, followed by α-ZEL and AOH. In this alternative binding pocket, AS forms hydrophilic interactions with W336, R426, K433, FAD, S1225, K1228, and S1234, while α-ZEL showed hydrophobic interactions with L147, A338, I1229, and A1231. 

Notably, in our present investigations, FAD and Fe_2_S_2_ were included in XO, and their proper partial charge distributions were calculated. In the recent study of Fan et al. [35], another binding pocket of AOH was also suggested during the blind docking calculations; nevertheless, this earlier evaluation was performed with the exclusion of FAD and Fe_2_S_2_.

Considering the results of incubation assays, mycotoxin depletion experiments, and modeling studies, AOH, AS, and α-ZEL seem to be allosteric inhibitors of the XO enzyme. It is also supported by the previous report of Fan et al. [35], where the non-competitive inhibitory mechanisms of AOH and AME were described.

## 3. Conclusions

In summary, the effects of 31 mycotoxins were examined on XO-catalyzed uric acid formation. Based on the in vitro enzyme incubation assays, mycotoxin depletion experiments, and molecular modeling studies, AOH, AS, and α-ZEL proved to be moderate allosteric inhibitors of XO. Our results also demonstrated that AOH, AS, and α-ZEL are inhibitors but not substrates of the enzyme. The above-listed observations make the suitability of AOH as a leading compound in the development of new XO inhibitors questionable, even if the structural modification results in the decreased toxicity of a derivative. Considering the typically nanomolar concentrations of mycotoxins in the circulation, it is reasonable to hypothesize that AOH, AS, and α-ZEL are not able to produce a clinically relevant decrease in uric acid levels and cannot interfere with the pharmacokinetics of drugs biotransformed by XO (e.g., 6-mercaptopurine). Because α-ZEL is a frequent contaminant in bovine milk, it is reasonable to hypothesize that this mycotoxin partly appears in milk in XO-bound form. Our results promote the deeper understanding of mycotoxin-XO interactions.

## 4. Materials and Methods

### 4.1. Reagents

Alternariol-9-methylether (AME), aflatoxin B1 (AFB1), aflatoxin B2 (AFB2), aflatoxin G1 (AFG1), aflatoxin G2 (AFG2), sterigmatocystin (STC), cyclopiazonic acid (CPA), citrinin (CIT), ochratoxin A (OTA), patulin (PAT), deoxynivalenol (DON), fumonisin B1 (FB1), T-2 toxin (T2), zearalenone (ZEN), α-zearalenol (α-ZEL), β-zearalenol (β-ZEL), zearalanone (ZAN), α-zearalanol (α-ZAL), β-zearalanol (β-ZAL), xanthine oxidase (XO; from bovine milk), xanthine, uric acid, and allopurinol were from Merck (Darmstadt, Germany). Alternariol (AOH), beauvericin (BEA), ochratoxin B (OTB), and ochratoxin C (OTC) were purchased from Cfm Oskar Tropitzsch GmbH (Marktredwitz, Germany). Alternariol-3-sulfate (AS), alternariol-3-glucoside (AG), alternariol-9-methylether-3-sulfate (AMS), alternariol-9-methylether-3-glucoside (AMG), zearalenone-14-sulfate (Z14S), and zearalenone-14-glucoside (Z14Glz) were obtained from ASCA GmbH (Berlin, Germany). Aflatoxin M1 (AFM1) and dihydrocitrinone (DHC) were from Apollo Scientific (Cheshire, UK) and AnalytiCon Discovery (Potsdam, Germany), respectively. Stock solutions of xanthine (1 mM, in DMSO) and uric acid (2 mM, dissolved in 0.01 M sodium hydroxide) were prepared and stored at −20 °C. Stock solutions of mycotoxins (5 mM or 10 mM) were prepared in ethanol or in dimethyl sulfoxide (DMSO).

### 4.2. Xanthine Oxidase Assay

The in vitro XO assay was carried out as was previously reported [41,42]. Xanthine (5 μM) was incubated with XO (0.0012 U/mL) enzyme in the absence and presence of increasing concentrations of mycotoxins (0.00, 0.10, 0.25, 1.0, 5.0, 10, 20, 35, 50, and 100 μM) in a thermomixer (5 min, 700 rpm, 37 °C). Incubates were prepared in sodium phosphate buffer (0.05 M, pH 7.5) with 500 μL final volumes. Solvent controls were tested in each experiment. As a positive control inhibitor, the impacts of allopurinol (0.000, 0.002, 0.010, 0.10, 0.50, 1.0, 2.0, 5.0, and 20 μM) were also examined. Incubations were started with pipetting XO solution to the samples, after which the enzyme reaction was stopped with 30 μL of 6 M HClO_4_. Samples were vortexed, then 97 μL of 1 M potassium hydroxide solution was added. After cooling (to 3 °C) and centrifugation (5 min, 12,000 rpm, 3 °C), concentrations of xanthine and uric acid was directly quantified from the supernatant by HPLC-UV (see details in Section 4.4).

The same experimental design was applied with the following modifications to test the reversibility of the mycotoxin-induced inhibition of XO. In these experiments, incubates contained mycotoxin (50 μM), the enzyme (0.0012 U/mL), and increasing concentrations of xanthine (5, 10, or 25 μM). XO was preincubated (10 min, 700 rpm, 37 °C) with the mycotoxins; thereafter, the incubation (5 min, 700 rpm, 37 °C) was started with the addition of xanthine. Other experimental details remained unchanged.

After the concentrations of uric acid (*c_uric acid_*) and xanthine (*c_xanthine_*) were quantified in the samples, we calculated the rate of metabolite formation (*R*).

*R* (%) = 100 × c_uric acid_ / (*c_uric acid_* + *c_xanthine_*), (1)

Then the *R* values of control samples were used as the bases of comparison (100%) when the inhibitory actions of mycotoxins were examined:

Uric acid formation (%) = 100 × *R_inhibitor_* / *R_control_*, (2)

where *R_inhibitor_* and *R_control_* are the metabolite formation rates in the presence and absence of the inhibitor, respectively. IC_50_ values were determined with sigmoidal (Hill1) fitting employing the OriginPro 8 program using these data (OriginLab Corporation, Northampton, MA, USA).

### 4.3. Mycotoxin Depletion Assays

In order to test the potential XO-catalyzed biotransformation of AOH, AS, and α-ZEL, these mycotoxins (each 5 μM) were incubated with XO enzyme (0.0012 U/mL) for 0 min, 30 min, and 60 min. Incubations were performed in the absence of xanthine. Other experimental details were the same as described in Section 4.2. Mycotoxin levels in the supernatants were quantified with HPLC-FLD (see details in Section 4.4).

### 4.4. HPLC Analyses

HPLC measurements were carried out using an integrated HPLC system (Jasco, Tokyo, Japan) containing a binary pump (PU-4180), an autosampler (AS-4050), a UV detector (UV-470), and a fluorescence detector (FP-920). Chromatograms were evaluated with ChromNAV2 software (Jasco).

The quantitative analyses of xanthine and uric acid were performed applying the following HPLC-UV method [41]. A Security Guard (C18, 4.0 × 3.0 mm; Phenomenex, Torrance, CA, USA) precolumn and a Kinetex EVO C18 (250 × 4.6 mm, 5 μm; Phenomenex) analytical column were used (isocratic elution; room temperature; flow rate: 1 mL/min; injected sample volume: 20 μL; detection: 275 nm). The mobile phase contained sodium phosphate buffer (10 mM, pH 4.55) and methanol (98:2 *v*/*v*%).

AOH was analyzed employing the following HPLC-FLD method [46]. A Security Guard (C18, 4.0 × 3.0 mm; Phenomenex) precolumn and a Kinetex EVO C18 (250 × 4.6 mm, 5 μm; Phenomenex) analytical column were used (isocratic elution; room temperature; flow rate: 1 mL/min; injected sample volume: 20 μL; detection: λ_ex_ = 335 nm, λ_em_ = 455 nm). The mobile phase contained acetonitrile and 1 mM phosphoric acid (35:65 *v*/*v*%). The same method was applied for the quantification of AS, except for the eluent used, which contained acetonitrile and 1 mM phosphoric acid (52:48 *v*/*v*%) [47].

The quantitative analysis of α-ZEL was performed by applying the following HPLC-FLD method [48]. A Security Guard (C18, 4.0 × 3.0 mm; Phenomenex) precolumn and a Kinetex EVO C18 (250 × 4.6 mm, 5 μm; Phenomenex) analytical column were used (isocratic elution; room temperature; flow rate: 1 mL/min; injected sample volume: 20 μL; detection: λ_ex_ = 274 nm, λ_em_ = 440 nm). The mobile phase contained water, acetonitrile, and methanol (46:46:8 *v*/*v*%).

### 4.5. Modeling Studies

The structures of AOH, AS, and α-ZEL were built in Maestro (Schrödinger, Maestro Schrödinger Release 2020-4). The energy minimization of the ligands was carried out with OpenBabel [49], using a steepest descent and a conjugate gradient algorithm. Gasteiger–Marsilli partial charges [50] were assigned to the ligand atoms in AutoDock Tools [51]. Flexibility was allowed on the ligands at all active torsions.

Atomic coordinates of XO were obtained from the Protein Data Bank (PDB) with PDB code 3eub [44], similar to our earlier study [41]. The amino acids of the target molecule were equipped with polar hydrogen atoms and Gasteiger–Marsilli partial charges in AutoDock Tools. The geometry and partial charges of the non-amino acid molecules, as the flavine-adenine dinucleotide (FAD), molibdopteroate, and the Fe_2_S_2_ inorganic cluster were calculated by MOPAC [52] with a PM7 parametrization [53], and a gradient norm of 0.001. The reduced form of FAD was used, according to Kuwabara et al. [45].

Ligands were docked to XO using AutoDock 4.2.6 [51]. The number of grid points was set to 126 × 126 × 126 at a 0.850 A grid spacing. A blind docking [54,55] investigation was carried out, where the docking box covered the whole surface of the target molecule. The Lamarckian genetic algorithm was used for the global search. A hundred docking runs were executed for each ligand, and the resulting ligand conformations were ranked by their free energy [56]. The lower rank means the higher calculated free energy (ΔG_binding_). The docked ligand conformations demonstrated were employed for subsequent evaluations [57].

### 4.6. Statistical Analyses

The mean and standard error of the mean (± SEM) values are demonstrated in the figures and tables. Statistical evaluations (*p* < 0.05 and *p* < 0.01) were executed by applying one-way ANOVA and Tukey’s post hoc test with SPSS Statistics software (IBM, Armonk, NY, USA).

## Figures and Tables

**Table 1:** Most important properties of mycotoxins and mycotoxin metabolites that were examined.

Mycotoxin	Abbreviation	Occurrence	Fungi	Toxic Effects	Reference
Alternariol Alternariol-9-methylether Alternariol-3-sulfate Alternariol-3-glucoside AME-3-sulfate AME-3-glucoside	AOH AME AS AG AMS AMG	Tomatoes, grapes, and corresponding products	*Alternaria* sp.	Endocrine disruptor and carcinogenicity	[5,6,7,8,9]
Aflatoxin B1 Aflatoxin B2 Aflatoxin G1 Aflatoxin G2 Aflatoxin M1	AFB1 AFB2 AFG1 AFG2 AFM1	Cereals, nuts, figs, vegetables, meat, milk, and dairy products	*Aspergillus* sp.	Hepatotoxicity	[10,11]
Sterigmatocystin	STC	Grains, coffee beans, cheese, spices, and soybeans	*Aspergillus* sp.	Hepatotoxicity and nephrotoxicity	[12]
Cyclopiazonic acid	CPA	Oilseeds, cereals, meat, and milk	*Aspergillus* sp. *Penicillium* sp.	Gastrointestinal toxicity and neurotoxicity	[13]
Citrinin Dihydrocitrinone	CIT DHC	Grains, rice, fruits, and spices	*Aspergillus* sp. *Penicillium* sp. *Monascus* sp.	Nephrotoxicity	[14,16]
Ochratoxin A Ochratoxin B Ochratoxin C	OTA OTB OTC	Cereals, fruits, meat, dairy products, and beverages	*Aspergillus* sp. *Penicillium* sp.	Nephrotoxicity	[17,18,19]
Patulin	PAT	Apple, pear, and corresponding products	*Aspergillus* sp. *Penicillium* sp.	Gastrointestinal toxicity and immunotoxicity	[15]
Beauvericin	BEA	Cereals and corresponding products	*Fusarium* sp.	Low toxicity	[20]
Deoxynivalenol	DON	Cereals and corresponding products	*Fusarium* sp.	Gastrointestinal toxicity	[20]
Fumonisin B1	FB1	Cereals and corresponding products	*Fusarium* sp.	Neural tube defects	[20]
T-2 toxin	T2	Cereals and corresponding products	*Fusarium* sp.	Gastrointestinal toxicity and toxic aleukia	[20]
Zearalenone α-Zearalenol β-Zearalenol Zearalanone α-Zearalanol β-Zearalanol Zearalenone-14-sulfate Zearalenone-14-glucoside	ZEN α-ZEL β-ZEL ZAN α-ZAL β-ZAL Z14S Z14Glz	Cereals and corresponding products	*Fusarium* sp.	Xenoestrogen, endocrine disruptor	[20,21,22]

**Table 2:** AOH, AS, and α-ZEL levels (% ± SEM) in samples after 0 min, 30 min, and 60 min incubation with XO (0.0012 U/mL; initial mycotoxin concentration: 5 μM; n = 3). We started the reaction with the addition of XO.

Incubation Time (min)	AOH Concentration (%)	AS Concentration (%)	α-ZEL Concentration (%)
0	100.0 ± 1.0	100.0 ± 0.1	100.0 ± 0.2
60	102.0 ± 0.9	100.3 ± 1.4	98.9 ± 0.9
120	96.4 ± 1.0	99.9 ± 1.0	100.0 ± 0.8

## Data Availability

Data will be made available upon request.
